# Safety and Benefits of Contraceptives Implants: A Systematic Review

**DOI:** 10.3390/ph14060548

**Published:** 2021-06-08

**Authors:** Morena Luigia Rocca, Anna Rita Palumbo, Federica Visconti, Costantino Di Carlo

**Affiliations:** 1 Operative Unit of Obstetric and Gynaecology, Pugliese-Ciaccio Hospital, 88100 Catanzaro, Italy; 2 Department of Obstetrics and Gynaecology, “Magna Graecia” University, 88100 Catanzaro, Italy; a.palumbo34@gmail.com (A.R.P.); fed.visconti@gmail.com (F.V.); cdicarlo@unicz.it (C.D.C.)

**Keywords:** contraception, etonogestrel, implanon, LARC, levonorgestrel, nexplanon, subdermal implant

## Abstract

Progestin-only contraceptive implants provide long-acting, highly effective reversible contraception. We searched the medical publications in PubMed, CENTRAL, and EMBASE for relevant articles on hormonal implants published in English between 1990 and 2021. Levonorgestrel (LNG) 6-capsule subdermal implants represented the first effective system approved for reversible contraception. The etonogestrel (ENG) single rod dispositive has been widely employed in clinical practice, since it is a highly effective and safe contraceptive method. Abnormal menstrual bleeding is a common ENG side effect, representing the main reason for its premature discontinuation. Emerging evidence demonstrated that it is possible to extend the use of the ENG implant beyond the three-year period for which it is approved. The ENG implant could be an effective and discrete alternative to the IUD in young girls, such as post-partum/post-abortion. Implants should be inserted by trained skilled clinicians who previously provide adequate counselling about their contraceptive effect, benefits, and any possible adverse events. More studies are needed to validate the extended use of the ENG implant for up to 5 years.

## 1. Introduction

Unplanned pregnancies (UPs) negatively affect the health system, since they not only lead to high social costs due to maternal and/or foetal morbidity, but also to the costs related to legal abortion [1].

Contraceptive systems represent a safe and effective instrument allowing fertile women who do not desire pregnancy to avoid it, and all women asking for contraception should receive detailed counselling about contraceptive choice [1].

Short-acting contraceptive methods (patch, vaginal ring, or oral contraceptives (OCs)) are characterized by impaired adherence, in terms of poor compliance and/or improper intake [2], with secondary relatively high UP risk.

Conversely, long-acting reversible contraceptives (LARCs) (intrauterine devices (IUDs), copper (Cu)-IUD, and subdermal contraceptive implants) provide at least 3-year continuous pregnancy protection and do not require any attention by users [1,2]. LARCs offer reversible long-term contraception, characterized by high continuation rates [3]. Therefore, they are excellent strategies to prevent UPs in all women not desiring a future pregnancy and not wanting a permanent contraception procedure [4].

Since 1991, when they were first introduced in the United States, progestin-only subdermal implants have become a safe, widespread contraceptive option [5], providing long-acting, highly effective reversible contraception [6].

The aim of the current review is to describe the available evidence about contraceptive implants in the clinical practice, in terms of safety, efficacy, and adverse events (AEs) in reproductive-aged women; furthermore, we aimed to evaluate the main evidence about their non-contraceptive effects (Table 1).

## 2. Evidence Acquisition

**Figure 1:**
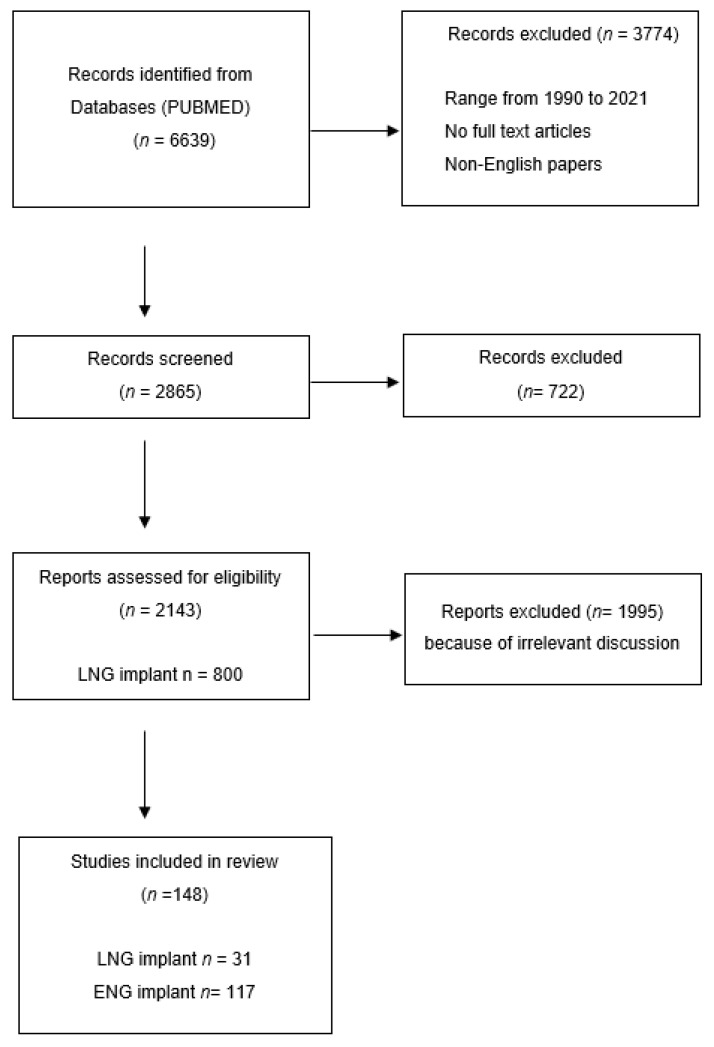
Flow diagram of study selection.

The present review has been conducted by a systematic study of scientific publications published from 1990 to 2021 about “contraception” and “implant” in women of reproductive age (adolescence, pre-menopause). We searched the following keywords: “contraceptives”, “contraceptive implant”, “etonogestrel”, “levonorgestrel”, “LARC”, “implanon”, and “nexplanon” in the PubMed database (Figure 1).

In this review, after a brief description of the main mechanism of action, we report the literature evidence regarding subdermal implants in terms of contraceptive efficacy and safety; then, we detail the available evidence about implant use in well-selected categories (adolescents/young women and post-abortion/post-partum or breastfeeding women); finally, non-contraceptive effects of subdermal implants are described.

## 3. Results

Subdermal implants are a progestin-only (etonogestrel (ENG), levonorgestrel (LNG)) contraceptive consisting of polymer capsules or rods placed under the skin that ensure a slow stable hormone delivery bypassing the first-pass hepatic metabolism [1,7,8]; they do not contain any oestrogens and do not induce plasma progestin peaks [9].

Implants should be inserted by trained skilled healthcare providers, and insertion should be preceded by adequate counselling about the contraceptive effect, benefits, and possible AEs. Only trained clinicians who completed the “Merck Clinical Training Program” are authorized to purchase Nexplanon. The Clinical Training Program for Nexplanon is offered to all eligible health-care providers in a live, two-hour, hands-on workshop. The duration of the implant depends on the progestin type and the polymer employed [7]. Once inserted, the device exerts a highly effective contraceptive action, with a prompt return to fertility after its removal [7]. Implants act by ovulation suppression, thickening of cervical mucus, and, lastly, inducing endometrial atrophy [10,11].

### 3.1. Levonorgestrel Implant

Norplant R represented the first effective system approved for reversible contraception [7]; it is an LNG six-capsule subdermal implant, and each capsule contains 36 mg of LNG. It placed under the skin of the upper arm during the first 7 days after menstruation, inducing a gradual plasmatic LNG release for about 5 years, regardless of user compliance [12,13]. Norplant delivers 50–80 mcg of LNG/day during the first year and 30–35 mcg for years 2–5 [14]. After the 5th year, 69% of the drug still remains in the capsules, ensuring contraceptive safety for women who delay implant replacement [15].

The main LNG contraceptive effect is due to a decrease in luteinizing hormone (LH) and follicle-stimulating hormone (FSH), with secondary ovulation suppression [14,15]; furthermore, Norplant reduces the rate of ovum transfer in the tube; at the endometrial level, LNG causes the inadequate development of the secretory endometrium, making it incompatible for implantation, while making the cervical mucus too thick and scanty, interfering with sperm migration [1,12,14,15,16]. Contraindications to its insertion are abnormal uterine bleeding, active liver disease, confirmed or suspected pregnancy, breast cancer, cancer of the genital tract, and cerebrovascular or coronary artery disease [14,16].

More than 60,000 women were enrolled in clinical trials evaluating Norplant efficacy [9]. Several studies showed a pregnancy rate in Norplant users of 0.6/100 woman-years after 1 year, while the 5-year cumulative pregnancy rate is 1–3.9/100 woman-years [9,16].

The LNG implant has a failure rate lower than OCs and IUDs [16] and, compared to OC and IUD, Norplant users have lower one-year pregnancy rates (0.6/100 vs. 2.3/100 vs. 2.4/100 for LNG implant, OCs, IUD, respectively) [14]. Norplant efficacy is inversely proportional to patient weight, so higher body mass index (BMI) was related to higher pregnancy risk [13].

The ectopic pregnancy rate is about 1.47/1000 Norplant users [14,17]. Interestingly, LNG implants had an ectopic pregnancy incidence lower than OCs, while no significant difference had been detected in the comparison with Cu-IUD users [9]. Compared to no contraception, the ectopic pregnancy rate was 0.30, 0.68, and 0.13 per 1000 women-years, in Norplant users, copper IUDs users, and women who have undergone bilateral salpingectomy, respectively [18].

In LNG implant users, the first year and fifth year continuation rates are 75–90% and 25–78%, respectively [16]. Interestingly, after 12 months, a higher continuation rate was observed in comparison with OC users (80% vs. 50%, respectively) [14]. Norplant acceptability, continuation, and tolerance rate were independent of patient age [13,19].

Compared to OCs, at one year, a significant (*p* < 0.001) higher continuation rate in implant users was observed (83.4% vs. 64.4% for LNG implant and OCs, respectively); similarly, a higher proportion of “very satisfied” users was noted in the implant group (28.5%) compared to the combined pill group (14.6%); in all enrolled subjects, discontinuation was due to AEs (such as menstrual changes and headaches) [20]. In several studies, discontinuation rates were 2% to 15% during the first year of use, and cumulative 5-year discontinuation rates ranged from 22 to 64 per 100 women [13]. In 1401 Nigerian LNG implant users, the main reason for early implant discontinuation was the wish to become pregnant (41.2%), while irregular bleeding was the third cause of discontinuation (11.3%) [21].

The main Norplant side effects are irregular menstrual bleeding especially within six months of insertion (increased or decreased menstrual flow, spotting, irregularity, and amenorrhoea), mood changes, and headaches [14]; thereafter, the gradual decrease in LNG release allows ovulatory-like cycles to return; thus, irregular bleeding diminishes over time [22]. Bleeding irregularities happen in 4.2–30.7% of users [13] and represent the main reason for discontinuation [16]. According to Fraser et al. [23], 12 months after implant placement, most women experienced prolonged bleeding at irregular intervals, while less frequently, patients had regular bleeding (25%) or amenorrhea (10%) [23]. In the following year, a progressive decrease in days and length of irregular bleeding was observed, so that during fifth-year use, 66% of women reported regular bleeding patterns [24]. In comparison with depot medoxyprogesterone acetate (DMPA), in LNG implant users, a higher average number of bleeding/spotting days was detected; interestingly, at 12 months, normal menstrual patterns were experienced by 23% of LNG implant users compared to 11% of DMPA users [25]. Some pharmacological strategies have been introduced in clinical practice in order to improve abnormal bleeding and reduce drug discontinuation. Indeed, oestrogen co-administration can reduce bleeding episodes but frequently leads to discontinuation due to gastrointestinal side effects [26]. Both mifepristone and tamoxifen seem to reduce irregular bleeding in Norplant users, but these findings need to be confirmed in larger trials [26].

Other Norplant side effects include skin reactions (including acne), dizziness, weight gain, breast discharge, and transient ovarian cysts [13,14,15]. In <1% of cases, infection at the insertion site was described [12]. The implant does not cause any major pathological changes in endometrium, liver, kidney, adrenal, or thyroid glands [16]. Furthermore, it induces only minor metabolic changes [17], without significant increase in glucose levels [14] and no significant changes in blood coagulation parameters, liver function, or lipid metabolism [14].

After Norplant removal, fertility promptly returns [9,16] within 3 months for 50% of users and within 1 year for 80% [14], without evidence of maternal and/or foetal adverse outcomes [17] as confirmed by toxicological and teratological data [16].

Norplant removal takes about 15–30 min to complete, and difficulties (non-palpable capsules, poorly positioned or misshapen capsules, heavy fibrous sheath encasing the capsules) may occur in about 13% of cases [12]. Non-palpable or partially palpable implants could be localized by using a 15–18 MHz linear array transducer; ultrasound is able to evaluate the relationship between implant, fascia, and neurovascular structures. Implant removal is easily performed under local anesthesia by using a modified vasectomy clamp through an incision of 5 mm or less. If subdermal dispositive could not be removed, a surgery is scheduled requiring the presence of an orthopedic surgeon [27].

Insertion site complications were usually a result of poor surgical technique (placing the capsules too superficially or too deeply); ulnar nerve neuropathy after insertion [28] or removal [29,30] was also observed.

Notwithstanding the high contraceptive efficacy of Norplant, its irregular bleeding and hazardous removal motivated researchers to develop a simpler subdermal dispositive [9]. Norplant II (Jadelle) consists of two silastic rods, each containing 75 mg LNG, that ensure 5-year-contraceptive protection [1,11,30]. The presence of only two silastic rods makes Jadelle a highly manageable system, characterized by simpler insertion and removal than Norplant, without any difference in serum LNG release [31,32]. Sino-implant (II) is a two-rod implant, releasing 150 mg of LNG; its mechanism of action is the same as Jadelle. Four randomized trials evaluated 15,943 Sino-implant (II) users, showing a 12-month pregnancy rate of 0.0–0.1% [33].

In 1998, Sivin et al. [34] in a prospective randomized trial compared Jadelle and Norplant users; interestingly, no pregnancies occurred in the first 4 years. At the 5-year follow-up, no significant difference in pregnancy rate (0.13% vs. 0.09% women-years for Jadelle and Norplant, respectively), cumulative continuation rate (55.1% vs. 53.0% for Jadelle and Norplant, respectively), or cumulative discontinuation rates was detected. In all patients, menstrual disturbances represented the main discontinuation reason; the only difference between implants was the mean removal time, which was significantly (*p* < 0.0001) shorter in Jadelle users (4.84 min) than in Norplant group (9.59 min) [34].

French et al. [35] did not find any significant difference in terms of continuation rate in women using Norplant II or LNG-20IUD. However, a significantly higher rate of irregular bleeding (prolonged bleeding and spotting) was observed in the Norplant II group [35].

Irregular bleeding represents the most common AE; ovarian cyst formation (persistent ovarian follicles) was seen at 12 months of use in 14.6% of LNG implant (Jadelle) users [1,36]. The occurrence of ovarian cysts or persistent ovarian follicles in implant users is a common non-pathologic finding undergoing spontaneous regression with no need for other treatment [36].

Lower abdominal pain was observed in 7–23% of patients; tenderness, numbness, tingling, and hyperpigmentation in more than 5%; and nausea, breast tenderness, loss of libido, and fatigue in about 2.9% [15]. As previously observed in Norplant users, fertility also promptly returns after removal of Norplant II [13], regardless of duration therapy or bleeding patterns [37].

### 3.2. Etonogestrel Implant

ENG is the main metabolite of Desogestrel (DSG), characterized by higher progesterone-like effects and lower androgen receptor affinity [1].

The ENG implant (Implanon (N.V. Organon, Oss, The Netherlands), Nexplanon (Merck, Kenilworth, NJ, USA)]) is a single rod containing ENG at the dose of 68 mg. It is placed under the skin of the upper non-dominant arm and left in place for three years [38]. ENG prevents LH release and hence ovulation; it thickens the cervical mucus and reduces the entry of spermatozoa and modifies the endometrium, inhibiting implantation of the fertilized ovum [38].

#### 3.2.1. Implanon

Implanon is a non-radiopaque single rod implant approved by the FDA in 2006 [6]. It acts through a slow and steady ENG release at a dose of 60–70 mg/day [39]. The average serum ENG level is 450 pg/mL, which decreases steadily to 200 pg/mL at the end of three years [40], ensuring contraceptive protection from 8 h until 3 years after placement [41]. Implanon is a subcutaneous contraceptive device consisting of a small plastic structure non-absorbable in the human body, about 4 cm long and 2 mm in diameter, containing 68 mg of etonogestrel. It has a small amount of barium sulfate in order to make it visible on X-ray [42].

Implanon inhibits ovulation by preventing the mid-cycle LH peak; initially, it suppresses follicular development and E2 production; after six months, ovarian activity slowly increases and FSH and E2 levels return to physiological values [43]. In the short term, this dispositive blocks ovarian function in almost 100% of cycles [15], while after 30 months, ovulation occurs in <5% of users. The physiological ovarian activity and the subsequent fertility return within 3–4 weeks after implant removal [43,44].

In the literature, Implanon’s contraceptive efficacy was widely recognized [9,43,45,46,47,48,49,50,51,52,53,54]. In a study by Croxatto et al. [45], no pregnancy during 53,530 cycles (4103 women-years) was observed [Pearl Index = 0.0 (95% CI, 0.00–0.09)], with a rapid ovulation return after its removal [45]. The same authors [43,44] evaluated the Implanon failure rate at three years follow-up; interestingly, no pregnancy (Pearl Index, 0; 95% CI 0.0–0.2) occurred during 1200 woman-years of exposure. Similarly, no pregnancies were reported in clinical trials after more than 70,000 cycles of use (Pearl Index = 0.0) [43,48,49,50,54]. In 2007, a Cochrane Review stated that no pregnancies were reported in Implanon in a group of 26,972 women [52]. Over a nine-year marketing period, in a study by Graesslin et al. [50], no in-treatment or pre-treatment pregnancies were reported, while fifty post-treatment pregnancies were observed (six within 14 days of implant removal), confirming that the implant achieves contraceptive protection exceeding 99% [53]. Similar results emerged from an integrated analysis conducted on 942 Implanon users; no pregnancy during treatment was observed, while six pregnancies occurred during the first 14 days after implant removal; thus, the cumulative Pearl Index was 0.38 [9].

The first case-report about ectopic pregnancy in reproductive-aged women users of Implanon was published in 2006 [55]; later, two other cases of ectopic pregnancy were described [56,57]. Implanon ectopic pregnancy could be due to the concomitant intake of drug-stimulating cytochrome P450, with a secondary increase in sex hormones’ metabolism and reduction of their contraceptive action [55]; higher BMI values were suggested as a possible predisposing factor [57], but no definitive risk factors have been identified yet [56].

Several studies confirmed that Implanon is a safe, well-accepted contraception method [46,47,49,54,58]. The main AEs related to ENG use are irregular periods, weight gain, acne, headache, breast tenderness, emotional lability, and abdominal pain [15]. During the first three months after positioning, infrequent bleeding is detected in about 50% of women, while in about 30% of users, prolonged bleeding was reported; amenorrhea occurs in 30–40% of cases [15]. After implant insertion, the characteristic of the bleeding pattern predicts the overall continuation rates; in fact, implant users with favourable bleeding are likely to continue long-term contraception [59]. According to Mansour et al. [59], a method to predict bleeding-related discontinuation consists of the evaluation of vaginal bleeding in any 90-day reference period. In particular, implant users with favourable bleeding (amenorrhea, infrequent bleeding, and normal frequency bleeding without prolonged bleeding) in the first reference period are likely to continue with favourable bleeding over the next 2 years. This method can facilitate counselling regarding bleeding for women using the ENG implant [59].

Croxatto et al. [46,47] showed that three-year Implanon use was a highly effective contraceptive option for women, with a discontinuation rate that decreased over the time (31% vs. 6% at 2- and 3-years follow-up, respectively) [46]; interestingly, bleeding irregularities represented the main reason for discontinuation [46,47]. Flores et al. [49], during an observation period that totalled 958.5 woman-years (27.5 months per woman), reported a continuation rate of 61.4% [49]. Funk et al. [60], in a multicenter clinical trial of 330 women, showed that common AEs leading to discontinuation, besides bleeding irregularities, were emotional lability (6.1%), weight increase (3.3%), depression (2.4%), and acne (1.5%) [60]. Blumenthal et al. [61] demonstrated that in a total of 942 women, the overall discontinuation rate was 32.7%; the most frequently reported reasons for discontinuation were AEs (13.9%), bleeding irregularities (10.4%), and planning pregnancy (4.1%); while headache (15.3%) was the most commonly reported drug-related complication [61]. In 2010, a prospective longitudinal study conducted on 32 reproductive-aged women showed an efficacy rate of 100% and a continuation rate of 93.8%; at six-month follow-up, a reduction in bleeding pattern was observed in 56.3%, while in 40.6% of patients, irregular bleeding was observed [62]. Conversely to previous papers [46], Modesto et al. [63] demonstrated that in ENG implant users, the discontinuation rate due to menstrual bleeding irregularities was 17% at 1 year and 62% at 2 years [63]. More recently, Nageso et al. [64] showed an Implanon discontinuation rate of 23.4%, with a mean duration of use of 9.6 ± 2.5 months; side effects represented the main reasons for discontinuation in 34.4% of cases [64]. In a retrospective study, Peterson et al. [65] documented a discontinuation of contraceptive method in 16% of implant users prior to 12 months, especially in women with irregular bleeding (odd ratio (OR) 4.36, CI: 2.71, 7.00) [65]. In a cross-sectional community-based survey [66] conducted on a total of 430 women, the overall discontinuation rate of Implanon was 34%; Implanon discontinuation was significantly observed in cases of women who never use a contraceptive method other than Implanon (OR 2.96, 95% CI 1.53–5.74), women who did not have a discussion with a partner (OR 3.32, 95% CI 1.57–7.04), poor counselling and follow-up (OR 9.23, 95% CI 4.7–18.13), fear of side effects (OR 0.12, 95% CI 0.058–0.24), and poor satisfaction of service (OR 5.2, 95% CI 2.77- 9.76) [66].

In order to reduce abnormal bleeding and the secondary drug discontinuation, clinicians usually prescribe OCs during implant use. Mifepristone combined with either E2 or doxycycline was significantly more effective than placebo in stopping bleeding in women with prolonged and/or frequent bleeding during Implanon use [67,68]. In a prospective cross-sectional study, Lazorwitz et al. [66] studied 350 healthy, reproductive-aged women using ENG implants for 12–36 months, showing that 20% received a prescription for OCs [69]. In a prospective randomized study [70], 84 Implanon users with prolonged or frequent bleeding were assigned to receive OCs containing Ethinlestradiol (EE2) 20 mcg/150 mg DSG for two cycles. A total of 32 women (76.2%) in the OC group and 15 women (35.7%) in the nonsteroidal anti-inflammatory drugs (NSAID) group stopped bleeding within 7 days after the initiation of treatment (*p* < 0.05). The mean duration of bleeding and spotting days in women treated with OC was significantly lower compared to the NSAID group (7.29 ± 3.16 vs. 10.57 ± 4.14 days (*p* < 0.05) [70]. Thus, OCs represent a manageable and effective treatment in order to control irregular bleeding in Implanon users.

Implanon metabolic effects have been evaluated in several studies. The implant does not exert a negative effect on cardiovascular risk factors (such as C-reactive protein (CRP), cholesterol/HDL ratio) [71,72] nor on carbohydrate metabolism [73,74,75,76]; furthermore, its use is related to lower risk of insulin resistance and dyslipidemia [76]. No negative impact on risk markers for atherosclerotic disease (such as IL-6, adiponectin, and Lp-a) was observed [72]; similarly, it does not exert a clinically relevant negative effect on endothelin-1 or TGF-beta [77]. Implanon does not significantly affect lipid profile or liver enzymes [6,78,79] and bone mineral density (BMD), even if its long-term use could negatively affect the mineral density at the distal radius and ulna [80].

Several studies compared Implanon to the LNG implant [52,81,82,83,84,85]. Affandi et al. [81] performed an integrated analysis of 13 different trials on 1716 Implanon users and 689 Norplant users; compared to the LNG implant, in Implanon groups, significantly fewer bleeding–spotting days (15.9–19.3 vs. 19.4–21.6; *p* = 0.0169), bleeding days (7.5–10.0 vs. 11.7–13.1; *p* < 0.001), and bleeding–spotting episodes (2.2–2.7 vs. 3.1–3.3; *p* < 0.0001) were observed, while no difference in discontinuation rates was detected [81]. In a prospective randomized trial, Mäkäräinen et al. [82] compared ENG vs. LNG implant users; no pregnancies were detected during the entire treatment period; ovulation restoration was observed at 18 months with Norplant and after 30 months with ENG implant; interestingly, a quicker Implanon removal time (5.9 +/− 3.4 min vs. 17.9 +/− 9.9 min for Implanon and Norplant, respectively) was observed [82]. In a randomized, multicentre trial, at 2-year follow-up, no difference in terms of failure rate (zero pregnancy) and restoration of fertility was detected; however, Implanon users not only had less frequent bleeding, but also quicker time (*p* < 0.001) of insertion and removal [83]. In a Cochrane Review by Power et al. [52], in the comparison of ENG vs. LNG implants, no difference in contraceptive effectiveness rates and continuation rate was observed; no pregnancies occurred. After two years, the amenorrhoea rate was significantly higher with Implanon. Bleeding pattern and menstrual changes were the most common side effect (*p* = 0.004), without significant (*p* = 0.17) difference between patients in AEs (acne, headaches, breast pain, increase in body weight); no significant difference in discontinuation rate due to AEs (6.0% vs. 7.6% for Implanon or Norplant, respectively) was also demonstrated. Finally, the authors confirmed that Implanon was significantly quicker to insert and remove than Norplant [52]. In a following multicentre trial by Meirik et al. [84], the quicker insertion of ENG implant than LNG subdermal dispositive was confirmed (51 vs. 88 s for ENG and LNG groups, respectively); however, at a six-week follow-up after insertion, no significant difference in terms of complication rates at the level of the insertion site was observed [84]. More recently, Okunola et al. [85] reported that 12 months after insertion, in the LNG group, weight gain was significantly higher than in the ENG group (3.16 ± 4.08 vs. 0.77 ± 3.76, *p* = 0.013; RR 1.69, 95%CI 1.46–1.96). The weight gain in the LNG group was in the range of −5.22 to 19.03 and in the etonogestrel group was in the range of −8.29 to 11.63 [85]. The mean weight difference in the levonorgestrel group was 3.16 (*p* = 0.004), while in the etonogestrel group it was 0.77 (*p* = 0.041); no difference in menstrual irregularities and client satisfaction was observed between groups [85].

When compared with the cu-IUD, the ENG implant showed a higher continuation rate both in the short [84] and long term [63]. In a randomized controlled trial (RCT) by Modesto et al. [63], at one year after placement, no significant difference in discontinuation rate due to menstrual bleeding irregularities was observed between women with the ENG implant and LNG-IUS (2.1% vs. 2.7% for ENG implant and LNG-IUS, respectively); the ENG implant and LNG-IUS presented similar continuation rates (82.6 vs. 81.0%), higher than the Cu-IUD (73.2%); the main reason for implant removal was weight gain [63]. In 2015, Berenson et al. [86] conducted a retrospective study on women who had LNG-IUS (n. 79,920) or ENG implants (n. 7374). LNG-IUS was more likely to be inserted than an ENG implant (*p* < 0.05). Abnormal menstruation represented the most frequent complication, which was more frequent in ENG implant users. The continuation rates were similar in both groups among teenagers, but ENG implants were more likely to be removed prematurely among women 20–24 years old (OR, 1.21; 95% CI, 1.06–1.39) and 25–44 years old (OR, 1.49; 95% CI, 1.35–1.64) [86].

#### 3.2.2. Nexplanon

Nexplanon (Merck Whitehouse Station, NJ, USA,) is a 4 cm rod-shaped radio-opaque ENG contraceptive containing barium, easily localized even if not palpable [11,38]. It is inserted subdermally in the inner non-dominant upper arm and should be removed no later than three years after insertion [5]; insertion and removal should be performed by a trained health care practitioner [87]. ENG is released at a rate of 35–45 mcg daily for the first year, 30–40 mcg daily for the second year, and 25–30 mcg daily at the end of the third year [87]. According to WHO medical eligibility criteria, it is contraindicated in women with a history of deep vein thrombosis, severe liver disease, or breast cancer [88].

Like the other ENG implant, Nexplanon is an effective contraception system [40,82,89], achieving suppression of ovulation at serum ENG level of 90 pg/mL [89]; notwithstanding a physiological reduction in progestin release, the plasmatic ENG level always results at contraceptive concentration (196 pg/mL after 12 months and 156 pg/mL at the end of the third year) [40]. In Nexplanon users, a full inhibition of ovulation in the first 24 months has been detected, whereas ovarian activity rarely occurs in the third year of use [82,90].

Nexplanon is a highly effective and safe contraceptive method, with a Pearl Index 0.0. In a three-year, no-comparative, multicentre study by Mommers et al. [91] in 301 reproductive-aged implant users, no pregnancy was detected; serious AEs were reported in 5.3% of women, even if none of these were directly related to the drug [91]. In December 2020, the Canadian Agency for Drugs and Technologies in Health [87] published a Clinical Review Report in order to evaluate Nexplanon’s contraceptive efficacy in healthy users. By review analysis, the overall Pearl Index was zero contraceptive failures per 100 woman-years; no pregnancy was observed also in women weighing >70 kg over three years [87].

Patients may choose to discontinue treatment if they desire pregnancy, are no longer sexually active, or no longer require contraception [84]. After implant removal, return of normal menses occurred in 83.5–94.4% of patients [87]. The review confirms a prompt and safe fertility return after Nexplanon removal, reporting seven pregnancies after the end of the contraception period [87]. Current evidence suggests that Nexplanon is not teratogenic, and there is no evidence that pregnancy with the implant in situ could be detrimental to woman, fetus, or pregnancy outcome [90,92,93].

Abnormal menstrual bleeding is a common ENG side effect, representing the main reason for its premature discontinuation [94]. In a retrospective observational study of 221 implant users, notwithstanding the well-recognized ENG effectiveness and tolerability, discomfort due to bleeding alterations/other AEs caused implant removal in 25.7% of women [95]. In the CADTH review [87], specific bleeding-related AEs (including dysmenorrhea, menorrhagia, metrorrhagia, vaginal hemorrhage, and genital hemorrhage) occurred in 3.8% to 46.2% of patients treated with the radiopaque etonogestrel implant [87].

By review results [87], the discontinuation rate accounted for 34.9–48.2% of patients; the main reasons for discontinuations were bleeding irregularities (amenorrhea, frequent irregular bleeding, heavy menstrual flow, spotting) (35.0–48.2%) and AEs (9.6–16.1%) [87].

There is no way to predict which women will develop bleeding disturbances, and there is no effective preventive treatment to avoid menstrual bleeding irregularities [63]. The basal weight was considered a predictor of irregular bleeding, but conflicting data are available. Casey et al. [96] found that obese women were 2.6 times less likely to remove the subdermal implant for bleeding vs. normal/overweight women (95% CI, 1.2–5.7; *p* = 0.014) [96]. Conversely, in a prospective observational study, Di Carlo et al. [97] found that lower basal BMI may account for the higher percentage of irregular bleeding [97]. Since unacceptable bleeding represents the most common cause of implant removal, medical interventions could improve irregular bleeding [98,99].

In a double-blinded RCT, the administration of OCs in ENG implant users induced a significant (*p* = 0.09) reduction of bleeding in 92% of patients [98]. In 2020, a double-blind RCT evaluated the short course of the tamoxifen effect on ENG- bleeding related; compared to placebo, at 90 days, the tamoxifen group reported an average of 9.8 (95% CI 4.6–15.0) more consecutive days of amenorrhea, higher total days of no bleeding (73.5 vs. 68 for tamoxifen and placebo, respectively, *p* = 0.001), and higher satisfaction rate (*p* = 0.023) [99]. Thus, in ENG implant users, OCs [98] or tamoxifen [99] could reduce bleeding and improve satisfaction, reducing the removal rate related to bleeding.

Other AEs secondary to implant positioning are emotional instability, weight increase, headache, acne, mood disturbances, nausea, lower abdominal pain, hair loss, loss of libido, pain at the implant site, neuropathy, and follicular cysts [5]; vascular disorders (such as deep vein thrombosis) were rarely described [87].

Nexplanon showed a low metabolic and bleeding impact, inducing no significant decrease in aspartate aminotransferase, alanine aminotransferase, cholesterol, triglycerides, or activated partial thromboplastin time [76,100,101]; no abnormalities in carbohydrate metabolism [75,101] and no changes in BMD were also observed [99]. Conversely, an increase in mean haemoglobin, haematocrit, and indirect bilirubin concentrations was detected [76,100]. Gain in body weight (+2.1/+4.1 kg at 12 months) [102,103] was demonstrated in 7.7–11.6% of users [87]. In the long term, the ENG implant was not associated with increased risk of thrombotic stroke and myocardial infarction [104] or of ovarian or breast cancer [105,106].

The Nexplanon Observational Risk Assessment (NORA) study demonstrated that the incidence of incorrect insertion was 12.6 per 1000 insertions (95% CI, 10.2–15.5) [107]. Other implant site complications are pain, infection (cellulitis or abscess), hematoma, abnormal scar development, and blood vessel injuries [5,108]; wound breakdown and subdermal implant reaction were also described [109]. Even if rare, three cases of allergic reaction to barium sulfate are also described in the literature [109,110,111]. Given the location of the implant in the medial antebrachial interval, there is a risk of neurovascular injury, especially with malpositioned or deeply placed implants. Reports of proximal median nerve injury leading to severe neuropathy [112] and acute ulnar nerve neuropathy [113] were published.

Nexplanon was palpable in 99.7–100% of patients and clearly visible in almost all women (96.2–100%) after insertion and before removal [87]. If the implant is not palpable, it should be localized and removed as soon as possible to prevent its migration [87]. The implant contains barium sulphate so it can be seen by high-frequency ultrasonography, X-ray, or computed tomography [5,41].

Real-world evidence demonstrated Nexplanon migration into pulmonary vasculature with an estimated incidence of 3.17 per 100,000 implants (95% CI, 1.37 to 6.24) [114]. Pulmonary embolization of the device is an iatrogenic condition that could present with symptoms such as chest pain or dyspnea [5], leading to respiratory issues and life-threatening conditions [87]. Several authors reported implant local migration to ipsilateral axilla [115,116] or to distant intravascular migration in the lobe pulmonary artery [5,90,108,116,117,118,119,120]. Migration could be solved with interventional radiology [105] or thoracoscopically by removing the implant from the pulmonary artery, avoiding, if possible, thoracotomy or lung resection [117]; in two cases, the migrated dispositive was left in place [120].

There are contradictory results from different studies regarding the association between sexual dysfunction and hormonal contraceptives [121]. Di Carlo et al. [122] demonstrated that in the short term (first three months of treatment), patients experienced a temporary reduction of vitality, mental health, social functioning, and emotional role functioning; thereafter, an improvement in general health status and physical role status was observed [122]. Some data suggested a positive Nexplanon influence on sexual function (increase in pleasure, personal initiative, and orgasm) with a secondary decrease in anxiety and discomfort [101]; conversely, other authors suggest that ENG implants had a negative influence on sexual function, probably by blocking ovarian function and thus reducing the production of androgens and oestrogens [123]. A rare case of secondary etonogestrel-related anorgasmia was described [124]. Indeed, if contraceptive-related female sexual dysfunction is suspected, contraceptive therapy should be discontinued [121].

Individuals demonstrated a wide variability in serum ENG concentrations, which can potentially affect side-effect profiles and efficacy [125]. The ENG implant may be a good contraceptive choice for obese women, since it does not increase the risk of thrombosis [126]. Even if obese ENG users have lower plasma progestin concentration [43,125,127], these serum concentrations are enough to consistently suppress ovulation [125,126]. Diet (*p* = 0.22) and exercise (*p* = 0.72) had no influence on serum ENG concentrations [128]. In the subanalysis of the Contraceptive CHOICE Project, at 3-year follow-up, the cumulative failure rate for the implant was less than one per 100 women-years, independent of BMI [129]. In a following Cochrane review [130], no relationship between higher BMI and effectiveness of hormonal contraceptives was identified. Interestingly, in healthy overweight women using the ENG implant, no major differences in carbohydrate metabolism were observed [130,131,132].

Compared to the non-radiopaque ENG implant, no difference in terms of contraceptive efficacy was detected (overall Peral Index was zero contraceptive failures per 100 woman-years) [87,133]; after removal, 94.4% of patients treated with the radiopaque ENG implant and 90.5% of patients treated with the non-radiopaque etonogestrel implant experienced return of menses to normal [87,133]; Nexplanon was always palpable in all patients (100%), while the non-radiopaque implant was palpable in 97.1% of users at 30 months [84,133]; finally, the radiopaque dispositive has a shorter insertion (27.9 s vs. 78 s) and removal time (119.3 vs. 228) than Implanon [87], inducing a real advantage in the clinical practice.

Several papers compared the ENG implant (Nexplanon) with the LNG implant [1,134,135,136,137] or LNG-IUD [38,63,138,139,140,141].

In 2015, in a multicentre RCT, Bahamondes et al. [134] demonstrated that ENG- and LNG-releasing implants are safe and highly efficacious contraceptives (pregnancy rates 0.0–0.5 per 100 women-years). No significant difference in discontinuation rates due to bleeding was observed between groups (6.7 vs. 12.5/100 women per year for the ENG implant and LNG implant, respectively). However, ENG users had lower 3-year cumulative loss to follow-up (8.1 vs. 14.4 per 100 women-years) and shorter duration of implant removal (*p* < 0.0001) [134]. The high contraceptive effectiveness of ENG and LNG implants was also confirmed in the long term [1,136,137], even if during the 3 years of follow-up all implant users had a small but significant weight increase [135].

In the comparison between LNG-IUS and ENG implants, at one-year follow-up, ENG implant users showed lower continuation rate and lower satisfaction rate [138,139,140], while a higher discontinuation rate was observed (19.6% vs. 26.8% for LNG-IUS 8 and ENG, respectively) [140]. After 12 months, fewer LNG-IUS users discontinued because of increased bleeding (3.2% vs. 11.3%) or AEs (14.3% vs. 21.8%) [140]. In 2021, Moray et al. [38] performed a meta-analysis of studies comparing the ENG implant with other contraceptive systems. Authors showed that the pooled 1-year continuation rate was 84.8% for LNG-IUS and 76.5% for the ENG implant, confirming that the continuation rates at the end of one year were higher for LNG-IUS compared to the implant [38].

Interestingly, at the 24-month follow-up, the cumulative continuation rate was 82% in the LNG-IUS group and 67% in the ENG implant group [139]; no significant difference in discontinuation rate was detected (13% vs. 17% for LNG-IUS and ENG implant, respectively) [139]; bleeding problems represented the main reason for discontinuation [139]. Similar results emerged in the following study that did not find a statistical difference in the 12-month discontinuation rate (24.9 vs. 24% for LNG-IUS 13.5 and ENG implant, respectively) [142]; a similar 2-year continuation rate between LNG-IUD and ENG implant users (77.8% vs. 75.9%) was observed [141]. More recently, a retrospective cohort study of 2026 nonsterilised women showed that at 2 years, the use of implants was more likely to be discontinued than LNG-IUD (cumulative discontinuation rate 24.2 vs. 33.3 per 100 women-years for LNG-IUD and implant, respectively) [143].

#### 3.2.3. ENG Implant Extended Use above Three Years

The subdermal contraceptive implant is a safe and effective long-term contraceptive system approved by the FDA for 3 years [144]. Pharmacokinetic data confirmed that at 3 years, ENG plasmatic concentration is sufficient to ensure contraceptive action [145]; moreover, emerging evidence demonstrated that it is possible to extend the use of the ENG implant beyond three years [1].

Preliminary studies [83,146] showed that after 4 years, the ENG implant was a safe, well-tolerated, and effective system, and no pregnancies were observed. The US-based contraceptive CHOICE study evaluated the contraceptive efficacy of two additional years of ENG implants and at least one additional year of LNG-IUD [137,144]. At one additional year of ENG use, zero pregnancies during the follow-up period were observed (estimated failure rate per 100 women-years). Conversely, in LNG-IUD users, one pregnancy was confirmed (failure rate 0.51 per 100 women-years). At 2 years of postexpiration follow-up (5 years post-insertion), no pregnancies in implant users were observed (failure rates 0). In LNG-IUD users, two pregnancies have been reported; the failure rate in the sixth year of use of LNG-IUD is calculated as 0.25 (95% confidence interval, 0.04–1.42) per 100 woman-years; the failure rate during the seventh year is 0.43 (95% confidence interval, 0.08–2.39) per 100 woman-years.

Interestingly, the serum ENG evaluation showed that median levels remain above the ovulation threshold of 90 pg/mL for women in all BMI classes [137,144]. Among implant users, the median ENG level was 207.7 pg/mL (range 63.8–802.6 pg/mL) at the time of method expiration, 166.1 pg/mL (range 67.9 25.0–470.5 pg/mL) at the end of the fourth year, and 153.0 pg/mL (range 72.1–538.8 pg/mL) at the end of the fifth year [144].

In 2016, Ali et al. [145] in a multicentre trial evaluated the extended ENG use for at least 5 years compared to the LNG implant (Norplant); in both groups, no pregnancies occurred during the additional 2 years of follow-up. No difference in 5-year pregnancy rates was observed between groups (0.6 per 100 woman-years vs. 0.8 per 100 woman-years for the ENG and LNG implant, respectively). ENG-users were more likely than LNG-users to experience heavy bleeding (*p* < 0.05). The median duration of the implant removal procedure was 64 secs shorter for the one-rod ENG implant. This study showed that the ENG and LNG subdermal implants have the same contraceptive effectiveness beyond 3 years up to 5 years with no major differences in occurrence of side effects [145]. More recently, Thaxton et al. [11] published a review of randomized and non-randomized trials about the extended use of progestin implants, concluding that to date few studies are available, so no definitive data could be obtained and more RCT are needed.

### 3.3. Contraceptive Implant in Adolescent Girls

Adolescent girls represent a high-risk population for UP; thus, the use of a highly effective contraceptive method has been strongly recommended [147,148].

Compared to the short-acting approach, long-acting systems (such as the IUD and subcutaneous implant) have higher efficacy, continuation, and satisfaction rates, without effects on long-term fertility [148]; based on these features, they are suggested as an effective strategy to prevent UP in young girls [148]. IUDs and implants can be inserted at any time after excluding an ongoing pregnancy [149]. The so-called “Quick Start” initiation consists of placing LARC during the visit rather than waiting for the next menstrual period [148]; this method improves short-term compliance without increasing breakthrough bleeding or other side effects [147].

Intrauterine devices (Cu-IUD and LNG-IUS) are safe first-line contraceptive choices for adolescents [150,151,152], characterized by low failure rates and high one-year continuation rates [152].

The ENG implant could be an effective and discrete alternative to the IUD in young girls, not requiring daily user action, and can be used if oestrogen is contraindicated [147]. Even if Nexplanon is licensed for women aged between 18 and 40 years, the WHO states that no contraception method should be contraindicated based on age alone [153].

Some papers evaluated the ENG implant continuation rate in adolescent girls [154,155,156,157]. Interestingly, in all available studies, at 12 months after ENG implant placement, high continuation rates were detected [154,155,156,157]. Berlan et al. [154] report that the device was removed prior to 12 months (mean length use 7.5 months) in 10.3% of cases. In the same year, Obijuru et al. [155] showed that at one-year follow-up, the continuation rate was 78%. Diedrich et al. [156] in a systematic review concluded that at 12 months, the continuation rate was higher in implant users than IUD users (84% vs. 74%) [156]. More recently, Buyers et al. performed a retrospective study on PCOS adolescent ENG users, showing a one-year continuation rate of 77% [157]. Only one study [155] evaluated the ENG implant continuation rate at 24 and 36 months; these authors found a continuation rate of 50% and 40% at 24 and 36 months, respectively [155].

In all studies, irregular bleeding represented the main cause of implant removal both in the short [154,155,156,157] and long term [155].

Long-term ENG use does not lead to significant weight gain in adolescents [158]; furthermore, it does not negatively affect cardiovascular risk and metabolic profile [100]. The ENG release implant is a safe and effective system in adolescents with cardiovascular conditions [159], such as in young girls with PCOS [157] or diabetes [160]. Thus, in young persons, ease of use, high efficacy, and high acceptability make the ENG contraceptive implant an important choice to prevent undesired pregnancy [161]. Interestingly, no adverse obstetric and fetal outcome has been detected after the inadvertent implant placement in a young girl during the first trimester of pregnancy [162].

### 3.4. Contraceptive Implant Placement in Post-Partum/Post-Abortion Women

During the post-partum period or after abortion, women are at high risk of undesired pregnancy [163]; thus, LARCs (IUD, subdermal implant) represent an effective and safe contraceptive option in order to prevent repeated unplanned pregnancies in all reproductive-aged women, especially among adolescents [147,164].

#### 3.4.1. Post-Partum Implant Placement

Post-partum birth control is usually scheduled at the six-week post-partum visit [165]; however, most women are sexually active by this time with a secondary risk of an undesired pregnancy [166]. The immediate post-partum contraception within 96 h of delivery and prior to discharge from the hospital may improve contraceptive prevalence and prevent UP [166].

Immediate or delayed post-partum ENG implant positioning represents an effective safe contraceptive strategy, characterized by very low pregnancy rates during the first year of use, even if very low-quality data are available [166].

Several literature papers evaluated the characteristics of the immediate or delayed post-partum ENG subdermal implant in childbearing age patients.

Gurtcheff 2011 et al. [167] showed that the six-month continuation rate of contraceptive implant use was 97.1% for immediate postpartum insertion and 95.7% for the delayed postpartum insertion group [167]. In a retrospective cohort study on 262 women by Wilson et al. [168], the immediate post-partum implant placement was related to a three-year cumulative continuation rate of 66.3%; interestingly, the continuation rates were highest among populations most vulnerable to rapid repeat and unintended pregnancies, such as adolescents [168]. Sothornwit et al. [166] in a Cochrane Review did not find any difference between immediate or delayed post-partum implant insertion in terms of the continuation rate at six months (RR 1.02, 95% CI 0.93 to 1.11) or 12 months (RR 1.04; 95% CI 0.81 to 1.34) [166]. Similarly, in an RCT, Vieira et al. [169] showed similar 12-month continuation rates regardless of the time of implant insertion (98% vs. 100% for immediate and delayed insertion, respectively, *p* = 0.99) [169].

Abnormal bleeding represented the main reason for implant removal, regardless of the time of placement [170]. According to the results of a Cochrane review [166], compared to delayed insertion, immediate postpartum implant users had a higher mean number of days of abnormal vaginal bleeding within six weeks postpartum (5.80 days, 95% CI 3.79 to 7.81) and a higher rate of other side effects in the first six weeks after birth (RR 2.06, 95% CI 1.38 to 3.06) [166]; no difference in heavy, irregular vaginal bleeding or associated severe cramping within 12 months (RR 1.01, 95% CI 0.72 to 1.44) was observed [166]. In a more recent RCT [169] comparing immediate and delayed post-partum implant placement, at 12-months follow-up, bleeding patterns were similar between groups; amenorrhea rates were high in both groups during the follow-up, while prolonged bleeding episodes were always unusual [169].

In a non-blinded RCT, Bryant et al. [171] compared immediate or delayed post-partum implant insertion in 96 adolescent and young women. Compared to delayed placement, in the immediate group, a higher 3-month continuation rate (*p* = 0.02) was observed (92% vs. 70% for immediate and 6-week group, respectively); moreover, no significant (*p* = 0.75) difference in 12-month continuation rate was detected (81% vs. 78% for the immediate and 6-week group, respectively) [171]. More recently, Barbieri et al. [172] published a cross-sectional study about ENG implant acceptance during the immediate postnatal period among adolescents/young women (average age was 19.5 years) during the COVID-19 pandemic; even if a high rate of women (76.2%) chose the implant as a postpartum contraceptive system, among them, 89.1% were unsatisfied after implant placement (*p* = 0.07) [172].

Satisfaction rates among post-partum LNG-IUD users were higher than post-partum implant users (100% vs. 72.2%, *p* < 0.05); lower discontinuation rates in IUD users were also detected (3.6% vs. 12.2%, in LNG-IUD and implant group, respectively). LNG-IUD placement had a better bleeding profile, even if no significant difference in AEs was detected among groups [173].

Study findings suggest moderate interference in normal weight [169] and overweight/obese women’s ability to lose gestational weight after implant insertion [174].

The insertion of ENG implant during the immediate postpartum period was not associated with differences in lochia duration [175] or alteration in the maternal metabolism and hemostatic system (blood pressure, maternal, BMI, waist circumference, complete blood count, CRP, IL-6, TNF-alpha, lipid profile, fasting serum glucose) [176,177]; similarly, no increased rate of venous thromboembolism [178] or negative impact on bone turnover [179] were observed.

Even if some data showed a small increased risk of post-partum anti-depressant use among women using the ENG implant [180], in a recent retrospective study, Drake et al. [181] showed that the postpartum depression rate was significantly lower for women initiating immediate postpartum implants compared to those choosing other methods (4.1% vs. 9.5%, *p* = 0.04) [181].

For lactating women, contraception choice is limited by concerns about hormonal effects on milk quality/quantity and hormone passage to the infant [182]. Ideally, the contraceptive should not interfere with lactation or infant growth, and the immediate initiation of hormonal methods should not disrupt the onset of milk production [182].

The ENG implant insertion seems safe not only for women, but also for newborns. No difference was detected in breastfeeding outcomes (lactogenesis stage II, lactation failure, milk composition) in women who inserted the ENG implant in early post-partum or after 4–8 weeks [167]. Similarly, ENG implant insertion immediately postpartum does not alter the volume of breast milk intake [183]. Thus, breastfeeding seems to not be affected by the hormonal system, and no negative effects have been reported [1]. A case report published in 2016 [184] showed a reduced weight gain in a breastfed infant of a mother receiving the implant 4 weeks after partum; more recently, Carmo et al. [185] in an open RCT observed no significant difference at 12 months among breastfed infants whose mothers underwent early or delayed post-partum insertion of the ENG implant [185]. In two Cochrane systematic reviews [166,182], the ENG-releasing implant effects on lactation and infant growth were investigated; no significant differences in breastfeeding duration, breast milk composition, or infant growth were observed after comparison with other hormonal contraceptives [182]; similarly, no difference in breastfeeding rate at six months between immediate and delayed post-partum implant insertion was observed [166].

Some studies compared breastfeeding outcomes after immediate postpartum initiation of the ENG subdermal implant with other LARCs; no difference in total duration of breastfeeding and in newborn growth and psychomotor development were detected between breastfeeding implant users and non-medicated IUD users [186]. More recently, Krashin et al. [187] compared breastfeeding outcomes after immediate postpartum initiation of ENG (n.28) or LNG (n. 112) contraceptive implants, showing a high 21-month breastfeeding continuation in both groups (100% vs. 93.2% for ENG and LNG implant users, respectively, *p* = 0.18) [187].

#### 3.4.2. Post-Abortion Implant Placement

Post-abortion LARC use has increased over the years [188]. Choosing a contraceptive method at the time of abortion may influence continuation, and many factors (race/ethnicity, past contraceptive use, feelings towards pregnancy, stress, weight) should be evaluated during counselling before any prescription [163,189].

ENG subdermal implant placement could be easily and safely scheduled after a surgical or medical abortion; as in post-partum women, the subdermal contraceptive placement could be performed immediately post-abortion or after an interval. The risk of discontinuation due to irregular bleeding represents the main concern among clinicians in women who receive an immediate insertion [190].

A few studies evaluated ENG implant after surgical abortion at 6- [191] and 12-months [190,192] after placement. In an RCT, Cowett et al. [191] compared the 6-month use rate of the ENG implant placed immediately after surgical abortion vs. placement 2–4 weeks post-procedure. The placement rate was 100% in the immediate group compared with 42.7% in the delayed group (*p* < 0.01). After completing the 6-month follow-up, the continuation rate was significantly higher in the immediate than the delayed group (93% vs. 63.3%, *p* = 0.002) [191]. Madden et al. [190] did not identify any significant difference in the 12-month continuation rate between women undergoing immediate postabortion (n. 141) or interval (n. 935) implant placement (81.5% vs. 82.8%, respectively, *p* = 0.54); frequent or irregular bleeding represented the main discontinuation reason in both groups (65.4% vs. 56.7% respectively) [190]. Similarly, in a study by Mark et al. [192], the overall ENG implant continuation was acceptable with similar rates for postabortion and interval placement [192].

A few studies compared the immediate ENG implant postabortion insertion with other contraceptive systems. In a retrospective cohort study on 26,858 patients undergoing surgical abortion, 25.4% received immediate post-abortion LARC (14.2% LNG-IUD, 4.2% Cu-IUD, and 7.0% ENG implant); multiparous women and women older than 35 years were more likely to initiate intrauterine or implantable contraception immediately after surgery; interestingly, during the years, an increasingly high number of women chose the implant (from 2.4% in 2012 to 8.7% in 2017) [193].

Piva et al. [173] showed higher satisfaction rates among post-abortion LNG-IUD users than in implant users (100% vs. 72.2%, *p* < 0.05). More implant users (*p* > 0.5) withdrew from contraception and had unfavourable bleeding profiles, while no difference in AEs rate was detected [173]. In 2020, an observational study by Caruso et al. [194] compared immediate post-abortion implant placement (61.4%) with a short-acting contraceptive (20%) or non-hormonal contraceptive treatment (18.6%). Before the end of the study period, in the implant group, no pregnancy was detected, while in 88.5% of control women not using hormonal contraception, unintended pregnancies were observed [194]. Interestingly, patients in the ENG implant group had a significantly (*p* < 0.0001) greater improvement of quality of life compared with the control group [194].

ENG releasing subdermal implants are also employed after medical abortion. However, no definitive data about the timing of LARC placement are available, since the implant can be inserted on the same day of mifepristone administration (immediate placement) or 2–4 weeks after abortion (delayed placement). A prospective observational study by Barros Pereira et al. [195] showed that the immediate implant placement does not reduce mifepristone action, and no significant difference in terms of efficacy was observed between groups (96.5% vs. 98.4% for immediate and delayed placement group, respectively); in the immediate implant insertion group, a higher 6-month continuation rate (73.7%) was observed, while only 16.1% of women in the delayed placement group chose to insert the implant [195]. In an RCT, Hognert et al. confirmed that an implant inserted on the day of mifepristone did not impair the efficacy of the drug compared with routine insertion at 2–4 weeks after abortion (efficacy of medical abortion in 94.2% vs. 96% of patients, in immediate and delayed placement group, respectively). Compared to delayed placement (71.6%), a significantly (*p* < 0.001) higher insertion rate in the immediate group (98.9%) was observed. At the 6-month follow-up, fewer women (*p* = 0.018) in the immediate group had become pregnant again (0.8%) compared to the routine group (3.8%) [196]. In an RCT, Raymond et al. [197] confirmed that the insertion of ENG implants on the day of mifepristone intake does not increase the risk of failure of the medical abortion procedure. The 6-month pregnancy rates did not significantly (*p* = 0.28) differ between groups (0.5% vs. 1.4% for immediate and delayed placement group, respectively); the satisfaction rate was higher in immediate group than in the delayed placement group (79% vs. 54% respectively, *p* < 0.001) [197].

In a retrospective study, Park et al. [198] demonstrated that the administration of a progestin-based contraceptive such as an ENG implant or DMPA injection on the same day as mifepristone for medical abortion did not alter the successful abortion rates [198]. In a recent systematic review and meta-analysis, Schmidt-Hansen et al. [199] showed that after abortion, the risk of subsequent unintended pregnancy was lower for patients treated with the etonogestrel implant simultaneously with mifepristone compared to delayed treatment both after three (risk ratio = 0.10; 95% CI, 0.01 to 1.94; *p* = 0.13) and six months (risk ratio = 0.22; 95% CI, 0.06 to 0.78; *p* = 0.02). Therefore, in women undergoing pharmacological pregnancy termination, contraceptive implant insertion should be offered on the day of mifepristone [199].

### 3.5. Non-Contraceptive Use of ENG Implant

Even if ENG implants were initially developed to prevent unplanned and unintended pregnancy, non-contraceptive benefits have been also identified [134]. An advantage of LARCs is that they are non-oestrogenic contraceptive methods, so they can be used safely by women with medical conditions like diabetes, hypertension, systemic lupus erythematosus, and endometrial hyperplasia or by women with a history of solid organ transplantation or current or past venous thromboembolism [1].

Unfortunately, poor information about these alternative benefits related to implant placement is available, and the use of subdermal implants for non-contraceptive indication is not frequent. In any case, users of the etonogestrel-releasing contraceptive implant have the benefits of a reduction of pain associated with endometriosis [200] and are also effective in women suffering from endometriotic ovarian cyst or rectovaginal (RV) endometriosis [201]. Subdermal implants seem to have a role in the regression of endometrial intraepithelial neoplasia [202].

#### 3.5.1. Endometriosis

Endometriosis is a chronic oestrogen-dependent gynaecological disease characterized by endometrial tissue outside the uterine cavity; catamenial and not catamenial pain (dysmenorrhea, chronic pelvic pain, deep dyspareunia, cyclical intestinal complaints) represent the main endometriosis-related symptoms [203].

The choice between medical or surgical treatment for endometriosis depends on the site and extension of the disease and on patients’ age and reproductive needs. The surgical approach can remove endometriotic lesions but is unable to exert a definitive long-term treatment, with high post-operative recurrence risk [204].

The hormonal approach plays a key role in suppressing ovulation, treating endometriomas at an earlier stage, and preventing disease relapse in the short and long term after surgery [205,206,207]. Hormonal formulations (progestogen alone or OCs) are the first-line medical treatment employed in the clinical practice [208].

Several studies confirmed that ENG-releasing contraceptive implants improve endometriosis-related pain and are not inferior to DMPA [209] and LNG-IUS [210] in endometriosis pain relief. In 2005, a case series study showed that ENG subdermal implants could represent a treatment option in women with severe symptoms related to pelvic endometriosis [208]. More recently, Niu et al. [211] confirmed that compared to the baseline, at the 24-month follow-up, ENG users had a significant (*p* < 0.05) decrease in pelvic pain and menstrual volume (*p* < 0.05) [211].

Subdermal implants are also effective in women suffering from endometriotic ovarian cyst or rectovaginal (RV) endometriosis. At six- and 12-month follow-up, ENG implants significantly decreased endometrioma-related dysmenorrhea and dyspareunia, with secondary improvement in quality of life, without any effect on cyst volume [201]. Ferrero et al. [212] demonstrated the efficacy of ENG-releasing implant in treating symptoms related to RV endometriosis (non-menstrual pelvic pain, deep dyspareunia, dysmenorrhea, dyschezia) with a high 2-year continuation rate (93.0%); interestingly, at 6-month follow-up, the volume of the RV nodules was significantly lower compared with the baseline; a further decrease was also observed at 12- and 24-month follow-up [212]. In ENG implant users, a reduction in biomarkers of endometriosis (CA-125, CD23, endometrial nerve fibre density) was also observed [213,214].

#### 3.5.2. Endometrial Action

The ENG released by the subdermal implant exerts a direct endometrial action by binding the progestin receptors in the endometrial tissue; moreover, it acts indirectly in the endometrium through its suppressive effects on the hypothalamic–pituitary–ovarian axis [215]. This complex combination of progestin action results in endometrial modification in terms of histology and induces modifications in the bleeding pattern [215].

The ENG-releasing implant reduces endometrial thickness without increasing cervical intraepithelial neoplasia or cervical carcinoma risk [216].

In 2019, Wong et al. [202] published a case report where the ENG subdermal implant was used for the treatment of endometrial intraepithelial neoplasia in a 36-year-old obese woman with abnormal uterine bleeding declining surgery; after implant insertion, a regression of endometrial intraepithelial neoplasia was observed [202].

## 4. Conclusions

LARCs have demonstrated greater efficacy in preventing unintended pregnancy among all women in comparison with short-acting methods [1,217], independent of age, parity, or BMI [1]. Progestin-only contraceptive implants provide long-acting, highly effective reversible contraception [6] without oestrogenic side effects [19]

LNG 6-capsule subdermal implants (Norplant R, Norplant-II) represented the first effective system approved for reversible contraception [7,73]. Compared to OC and IUD, the LNG implant showed lower failure rates [16] and one-year pregnancy rates [14]. Bleeding irregularities are the main reason for discontinuation and the most commonly reported side effects [16].

The ENG implants are single rods containing ENG at a dose of 68 mg [38]. Implanon is a non-radiopaque single rod implant; Nexplanon, which is bioequivalent to Implanon, is a 4 cm rod-shaped radio-opaque ENG contraceptive containing barium, easily localized even if not palpable [11,38]. It is a highly effective and safe contraceptive method, with a Pearl Index of 0.0 [84,88]. After implant removal, return of normal menses occurred in 83.5–94.4% of patients [87]. Abnormal menstrual bleeding is a common ENG side effect, representing the main reason for its premature discontinuation [87]. The evidence generally did not indicate an association between higher BMI or weight and effectiveness of hormonal contraceptives [130]. The implant may be offered as a first-line contraceptive method to any woman seeking a reversible and reliable birth control method independent of BMI [129].

Emerging evidence demonstrated that it is possible to extend the use of the ENG implant beyond the three-year period for which it is approved [1], even if further studies are needed [15].

ENG implant could be an effective and discrete alternative to the IUD in young girls, not requiring daily user action, and can be used if oestrogen is contraindicated [1,147].

Immediate postpartum implant insertion may increase uptake of long-acting reversible contraception and help reduce short interpregnancy intervals and unintended pregnancy [170]; offering the ENG implant to youths during the immediate postnatal period is evidence-based care [172]. The ENG implant insertion seems safe not only for women but also for newborns [1].

The ENG-releasing subdermal implant could be employed immediately after a surgical abortion [190,191,192] or on the same day of mifepristone assumption, without an increase in pregnancy risk compared to the delayed placement 4–8 weeks after the procedure [195,196,197,199]. It has been shown to be effective in improving symptomatic endometriosis with reduction in pain severity and menstrual symptoms (dysmenorrhea and dyspareunia) [201,210,211]; ENG therapeutic efficacy for pain relief is not inferior to other progestins [209,210].

Further studies need to confirm the efficacy of the ENG implant in endometrial intraepithelial regression.

In conclusion, ENG-releasing implants are effective contraceptive methods approved in all reproductive-aged women; they represent safe and effective systems even when inserted immediately post-partum or post-abortion. Implants should be inserted by trained skilled clinicians who previously provide adequate counselling about their contraceptive effect, benefits, and any possible adverse events. Counselling is crucial, and when intensive counselling has been given, 1-year continuation rates have reached 80–90% in users of the ENG implant [63]. Since abnormal bleeding represents the main reason for implant discontinuation, counselling before placement and throughout the method use is the best strategy to enable the users to understand and accept these minor effects and thus continue using the method [1]. More studies are needed to validate the extended use of the ENG implant for up to 5 years.

## Figures and Tables

**Table 1:** Comparison between different subdermal implants.

Subdermal Implants	Norplant	Norplant II (Jadelle)	Implanon	Nexplanon
Composition	LNG 6-capsule	LNG-2 silastic rods	ENG-single rod implant	ENG- rod-shaped radio-opaque contraceptive ENG containing barium
Dosage	each containing 36 mg LNG	each containing 75 mg LNG	containing 68 mg ENG	containing 68 mg ENG
Duration	5 years	5 years	3 years	3 years
Principal medical reason for removal	irregular menstrual bleeding	irregular menstrual bleeding	irregular menstrual bleeding	irregular menstrual bleeding
Trackable	NO	NO	NO	YES
Mean time taken to remove	9.59 min	4.84 min	2.18 min	<2 min

## Data Availability

Not applicable.
